# Metabotropic Glutamate Receptors 1 Regulates Rat Carotid Body Response to Acute Hypoxia *via* Presynaptic Mechanism

**DOI:** 10.3389/fnins.2021.741214

**Published:** 2021-10-05

**Authors:** Chaohong Li, Baosheng Zhao, Chenlu Zhao, Lu Huang, Yuzhen Liu

**Affiliations:** ^1^Henan Key Laboratory of Neural Regeneration and Repairment, Life Science Research Center, The First Affiliated Hospital of Xinxiang Medical University, Weihui, China; ^2^Department of Thoracic Surgery, The First Affiliated Hospital of Xinxiang Medical University, Weihui, China

**Keywords:** carotid body, hypoxia, glutamate, group I metabotropic glutamate receptors, DHPG

## Abstract

**Background:** The carotid body (CB) plays a critical role in oxygen sensing; however, the role of glutamatergic signaling in the CB response to hypoxia remains uncertain. We previously found that functional multiple glutamate transporters and inotropic glutamate receptors (iGluRs) are expressed in the CB. The aim of this present research is to investigate the expression of group I metabotropic glutamate receptors (mGluRs) (mGluR1 and 5) in the CB and its physiological function in rat CB response to acute hypoxia.

**Methods:** RT-PCR and immunostaining were conducted to examine the mRNA and protein expression of group I mGluRs in the human and rat CB. Immunofluorescence staining was performed to examine the cellular localization of mGluR1 in the rat CB. *In vitro* carotid sinus nerve (CSN) discharge recording was performed to detect the physiological function of mGluR1 in CB response to acute hypoxia.

**Results:** We found that (1) mRNAs of mGluR1 and 5 were both expressed in the human and rat CB. (2) mGluR1 protein rather than mGluR5 protein was present in rat CB. (3) mGluR1 was distributed in type I cells of rat CB. (4) Activation of mGluR1 inhibited the hypoxia-induced enhancement of CSN activity (CSNA), as well as prolonged the latency time of CB response to hypoxia. (5) The inhibitory effect of mGluR1 activation on rat CB response to hypoxia could be blocked by GABA_*B*_ receptor antagonist.

**Conclusion:** Our findings reveal that mGluR1 in CB plays a presynaptic feedback inhibition on rat CB response to hypoxia.

## Introduction

Oxygen sensing mediated by central and peripheral chemoreceptors is essential for survival of multicellular organisms. Carotid body (CB), a highly vascular tissue, is mainly a peripheral chemoreceptor located in the adventitia of the bifurcation of the common carotid artery. CB is usually organized in glomeruli clusters, which are composed of four to eight neuron-like glomus cells (type I cells) and one to two glia-like sustentacular cells (type II cells) (Ortega-Sáenz and López-Barneo, 2020). Type I glomus cells are tyrosine hydroxylase (TH)-positive O_2_ sensing cells, and their cytoplasm is rich in abundant synaptic vesicles that contain neurotransmitters and neuromodulators. Type II sustentacular cells that encase clusters of type I cells are glial fibrillary acidic protein (GFAP) positive cells and can be transformed into type I glomus cells under certain conditions (Platero-Luengo et al., 2014).

Evidence supports that the type I glomus cells, as presynaptic neuroendocrine cells, establish synapses with afferent sensory fibers of carotid sinus nerve (CSN) (as postsynaptic nerve endings) (Ortega-Sáenz and López-Barneo, 2020). There is general acceptance that transduction of the hypoxic stimulus in the CB involves closure of O_2_-sensitive K^+^ channels, which leads to membrane depolarization of type I cells. This process induces Ca^2+^-dependent release of neurotransmitters from type I cells; subsequently, the neurotransmitters act on specific postsynaptic receptors on CSN afferent fibers (Chang et al., 2015). It is established that these neurotransmitters [such as ATP, GABA, and acetylcholine (ACh)] in type I cells regulate CB oxygen sensing not only by acting on the corresponding ionotropic receptors of chemosensory nerve endings (Zhang et al., 2000, 2009; Rong et al., 2003; Varas et al., 2003; Alcayaga et al., 2007; Sacramento et al., 2019) but also by acting on non-ionotropic receptors of chemosensory nerve endings (such as ATP and ACh) (Alcayaga et al., 2000; Shirahata et al., 2004) or non-ionotropic receptors of type I cells themselves (such as GABA and ATP) (Fearon et al., 2003; Tse et al., 2012). Studies show that neurotransmitters acting on non-ionotropic receptors of type I cells provide feedback mechanisms to regulate the CB hypoxia response. Xu et al. (2005) found that ATP exert strong negative feedback regulation on CB hypoxia response by restricting the increase in [Ca^2+^]i during hypoxia or acting on P2Y receptors on type I cells to close the background ion channels. GABA was also demonstrated to play a negative feedback role by acting on GABA_*B*_ receptors located on type I cells to inhibit the hypoxia-induced release of ATP and ACh (Fearon et al., 2003). Although evidence supports numerous mechanisms by which hypoxia-triggered depolarization of type I cells leads to excitation of the CSN, the glutamatergic neurotransmitter system its expression in CB as well as its effect on the hypoxia-triggered excitation of the CSN has not been systematically explored.

In the central nervous system, glutamate is the major neurotransmitter. In the process of glutamatergic synaptic transmission, once an action potential depolarizes the presynaptic membrane, glutamate accumulated in presynaptic transmitter vesicles by the vesicular glutamate transporters (VGluTs) is released into the synaptic cleft by exocytosis and then acts on the corresponding postsynaptic receptors to transmit neuronal signaling. Excitatory amino acid transporters (EAATs) on adjacent glial cells scavenge glutamate from the synaptic cleft to terminate glutamate’s functions (Zhou and Danbolt, 2014; Reiner and Levitz, 2018). Glutamate receptors are divided into two families: ionotropic glutamate receptors (iGluRs) and metabotropic glutamate receptors (mGluRs) (Reiner and Levitz, 2018). Playing an important role in synaptic transmission/plasticity, iGluRs are ligand-gated ion channels, which once activated leads to a rapid ion flux, and are further divided into three main subfamilies: alpha-amino-3-hydroxy-5-methyl-4-isoxazole propionic acid receptors (AMPARs), *N*-methyl-D-aspartate receptors (NMDARs), and kainate receptors (KARs) (Traynelis et al., 2010; Reiner and Levitz, 2018). mGluRs belong to the G protein-coupled receptor family and are divided into group I mGluRs (GluR1/5), group II mGluRs (mGluR2/3) and group III mGluRs (mGluR4, mGluR6–8) (Niswender and Conn, 2010; Reiner and Levitz, 2018). mGluRs activate the G protein-coupled signal cascades leading to changes in synaptic function/excitability by inhibiting the presynaptic transmitter release (Sladeczek et al., 1993; Gereau and Conn, 1995) or by regulating the postsynaptic response (Sheng et al., 2017; Jin et al., 2018).

It is established that glutamatergic signaling functions not only in the central nervous system but also in the peripheral nervous system (Tremolizzo et al., 2012; Chen and Kukley, 2020). Multiple VGluTs, EAATs (Li et al., 2020), and iGluRs (Liu et al., 2009, 2018) have been demonstrated to exist in the rat CB. We found that exposure of rats to cyclic intermittent hypoxia (CIH) alters the expression of some of these molecules. iGluRs in the CB are functional because blockading with the NMDAR antagonist MK801 reduces endothelin-induced CSN activity (CSNA) (Liu et al., 2018). These data suggested that iGluR-mediated glutamatergic signaling might occur in the CB, but the information regarding the mGluRs expression in the CB and its physiological function in the CB reponse to hypoxia is unknown.

We previously demonstrated that group I mGluRs exist in human and rat adrenal glands and that mGluR1 is involved in hypoxia-induced upregulation of TH in cultured rat adrenal medulla (Fan et al., 2020). Both adrenal medulla and CB originate from the neural crest and function as peripheral chemoreceptors in acute oxygen sensing (Gao et al., 2019). Taken together, these observations led us to speculate that group I mGluRs might be present in the CB and are involved in the carotid chemoreflex. In this study, we examined the expression of group I mGluRs in the human and rat CB and its cellular localization in the rat CB by RT-PCR and immunostaining. Moreover, we investigated the physiological function of mGluR1 in rat CB response to acute hypoxia.

## Materials and Methods

### Human Carotid Body

Human surgical specimens were obtained from The First Affiliated Hospital of Xinxiang Medical University with consent from patients (grant approval number: 2016008). Human CB specimen was obtained from a patient with left CB paraganglioma, and human cerebral cortex tissue was obtained from a patient with craniocerebral trauma. The protocol related to human subjects was conducted in accordance with the declaration of Helsinki and approved by the Human Ethics Committee of The First Affiliated Hospital of Xinxiang Medical University.

### Animals and Rat Carotid Body Harvest

Adult male Sprague-Dawley (SD) rats were ordered from Beijing Vital River Laboratory Animal Technology Co., Ltd. (Beijing, China). The age of the rats was 8 weeks, and the body weight was 240–250 g at entry into the protocol. Rats were housed under room temperature and standard humidity (50 ± 5%) with a 12-h day/night cycle with laboratory chow and water *ad libitum*. All procedures performed in this study were in accordance with national animal research regulations, and all animal experimental protocols were approved by the Institutional Animal Ethics Committee at The First Affiliated Hospital of Xinxiang Medical University.

After the SD rat was anesthetized by inhalation of 2% isoflurane (RWD Life Science, Shenzhen, China), the rat was decapitated and the bilateral carotid bifurcations were rapidly removed and placed into 95% O_2_–5% CO_2_ saturated ice-cold phosphate-buffered saline (PBS). The CBs were dissected (within 4 min) and immediately soaked in RNAlater (Qiagen, Valencia, CA, United States), then stored at −80°C until analyzed.

### RNA Extraction and Reverse Transcription-PCR

Total RNA was extracted from human CB specimens and rat CBs (total 16 CBs pooled from 8 rats) using TRIzol reagent (Thermo Fisher Scientific, Waltham, MA, United States) according to the manufacturer’s protocol. After removal of gDNA, 500 ng of total RNA was reversely transcribed into cDNA by using QuantiTect Reverse Transcription Kit (Qiagen, Valencia, CA, United States), in accordance with the manufacturer’s protocol. The RNA without reverse transcriptase was used in reverse transcription (RT) reaction to obtain a negative cDNA control. The mRNA expression level was detected by semi-quantitative PCR in a Veriti^®^ 96-well Thermal Cycler (Applied Biosystems, Foster City, CA, United States) using HotStarTaq^®^ Master Mix Kit (Qiagen, Valencia, CA, United States). Two microliters of cDNA samples was mixed with 10 μl of HotStarTaq Master Mix and 1 μl of gene-specific primer pairs in a final reaction volume of 20 μl to amplify the target gene. The PCR reactive conditions included an initial step at 95°C for 10 min, followed by 40 cycles at 94°C for 50 s, proper annealing temperature for 50 s, extension at 72°C for 1 min, and then ending at 72°C for 10 min. An equal volume of the negative cDNA control was used in the PCR reaction as negative PCR control. The PCR product was loaded into 1.2% agarose gel. A total of three technical replicates were conducted for each target gene. All primers were exon spanning and designed using Primer-BLAST.^1^ Details of all primers are listed in Tables 1, 2.

**TABLE 1:** Sequence of human primers used in RT-PCR experiment.

**Gene**	**Accession No.**	**Primer sequence**	**PCR Cycle no.**	**Annealing Tm (°C)**
*GRM*1	**NM_001278065.2**	F: 5′-CAGCCGATTCGCTTTAGCC-3′ R: 5′-GGGATCGCGGTACTGAAGTTG-3′	40	57
*GRM*5	**NM_000842.5**	F: 5′-TCCAGAATTTGCTCCAGCTT-3′ R: 5′-CTTCCATCCCACTTTCTCCA-3′	40	60
*GAPDH*	**NM_002046.7**	F: 5′-GCAGGGGGGAGCCAAAAGGGT-3′ R: 5′-TGGGTGGCAGTGATGGCATGG-3′	40	53

*GRM, glutamate metabotropic receptor; Tm, temperature; F, forward primer; R, reverse primer.*

**TABLE 2:** Sequence of rat primers used in RT-PCR experiment.

**Gene**	**Accession No.**	**Primer sequence**	**PCR Cycle no.**	**Annealing Tm (°C)**
*GRM*1	**NM_001114330.1**	F: 5′-CCAGTGATGTTCTCCATACC-3′ R: 5′-CACTCTGGGTAGACTTGAGTG-3′	40	50
*GRM*5	**NM_017012.1**	F: 5′-CCCCAAACTCTCCAGTCT-3′ R: 5′-ATTTTTCACCTCGGGTTC-3′	40	49
β*-actin*	**NM_031144.3**	F: 5′-GGGAAATCGTGCGTGACATT-3′ R: 5′-CGGATGTCAACGTCACACTT-3′	40	55

*GRM, glutamate metabotropic receptor; Tm, temperature; F, forward primer; R, reverse primer.*

### Immuno-Staining

After the rats were fixed with 4% neutral buffered formalin by cardiac perfusion, the carotid bifurcations containing the CB were removed and embedded in paraffin. The paraffin-embedded carotid bifurcations were cut by Shandon^TM^ Finesse^TM^ 325 Microtomes (Thermo Fisher Scientific, Waltham, MA, United States) to prepare CB sections (3 μm).

For immunohistochemistry staining, after being deparaffinized, hydrated, and antigen retrieved, the CB sections were blocked with 10% goat serum at room temperature for 1 h, then incubated at 4°C overnight with the following primary antibodies: rabbit anti-mGluR1 (1:300, Abcam, Cambridge, United Kingdom, Cat No. ab82211) and anti-mGluR5 (1:100, Abcam, Cambridge, United Kingdom, Cat No. ab76316). After being washed three times in PBS, sections were incubated with GTVision^TM^ III Detection System Mouse & Rabbit Kit (HRP/DAB) (GeneTech, Shanghai, China), according to the manufacturer’s instruction, to yield a brown crystalline antigen–antibody complex product. The paraffin-embedded rat brain sections were incubated with primary antibodies as the positive control. The negative staining control was prepared by replacing the primary antibody to PBS containing 5% normal goat serum. The staining was examined through the Nikon H600L microscope and photographed with Nikon digital camera DS-Fi1c (Nikon, Tokyo, Japan).

For double immunofluorescence staining, after blocking with 10% goat serum, the CB sections were exposed to a mixture of two primary antibodies as follows: rabbit anti-mGluR1 (1:100, CST, Danvers, MA, United States, Cat No. 12551) with mouse anti-TH (1:2,000, Sigma, St. Louis, MO, United States, Cat No. T2928); rabbit anti-mGluR1 (1:100, CST, Danvers, MA, United States, Cat No. 12551) with mouse anti-GFAP (1:200, CST, Danvers, MA, United States, Cat No. 3670). After washing with PBS, the sections were incubated with a mixture of second antibody of Alexa Fluor 488 goat anti-rabbit IgG (1:400, CST, Danvers, MA, United States, Cat No. 4412) with Alexa Fluor 555 goat anti-mouse IgG (1:400, CST, Danvers, MA, United States, Cat No. 4409). The negative staining control was prepared by omitting the primary antibody. The staining was examined and photographed with the Nikon C2 confocal microscope (Nikon, Tokyo, Japan).

### *In vitro* Carotid Sinus Nerve Discharge Recording

The CB response to hypoxia was recorded by *in vitro* CSN discharge recording similar to the method described in a previous report (Rong et al., 2003). Briefly, SD rat was anesthetized by inhalation of 2% isoflurane, and then decapitated. The bilateral carotid bifurcations containing the CB were then rapidly removed and placed into 95% O_2_–5% CO_2_ saturated ice-cold Kreb’s solution (in mM: NaCl 113, KCl 5.9, NaH_2_PO_4_ 1.2, MgSO_4_ 1.2, NaHCO_3_ 25, glucose 11.5, CaCl_2_ 2, and pH 7.4). The sinus nerve attached to the CB was dissected under a microscope, and the bifurcation was placed into a recording chamber (3 ml) perfused with 95% O_2_–5% CO_2_ saturated warm Kreb’s solution (15 ml/min) with the chamber temperature being kept at 33°C. The CSN discharge was recorded using a suction electrode (Warner Instruments, Hamden, CT, United States). The signaling was amplified (1,000×) and filtered (100–1,000 Hz) by a DP-311 differential amplifier (Warner Instruments, Hamden, CT, United States), then collected by PowerLab 8/35 (AD Instruments, Bella Vista, NSW, Australia) and analyzed and integrated by LabChart 8 software (AD Instruments, Bella Vista, NSW, Australia). To detect the CB response to hypoxia, the CB was exposed to 5% O_2_–5% CO_2_–90% N_2_ saturated Kreb’s solution for a period of 90 s at an interval of 15 min. Hypoxia-induced CSNA was normalized by integrated CSNA under hypoxia divided by the integrated baseline CSNA under 95% O_2_–5% CO_2_ condition. Group I receptor agonist (S)-3,5-dihydroxyphenylglycine (DHPG, 40 μM) (Sigma, St. Louis, MO, United States), mGluR1 antagonist (3,4-dihydro-2H-pyrano[2,3-b]quinolin-7-yl)-(cis-4-methoxycyclohexyl)-methanone (JNJ16259685, 20 μM) (Santa Cruz Biotechnology, Dallas, TX, United States), and DHPG (40 μM) combined with JNJ or DHPG combined with GABA_*B*_ receptor antagonist CGP52432 (10 μM) (Selleck Chemicals, Houston, TX, United States) were applied by switching the basal perfusate to 95% O_2_–5% CO_2_ saturated Kreb’s solution containing the compound for 10 min before switching to 5% O_2_–5% CO_2_–90% N_2_ saturated Kreb’s solution containing the compound.

### Statistical Analysis

The Shapiro–Wilk normality tests of all data could be conducted with the SPSS statistical software. All data were normally distributed, and the normality test results were shown in the online supplementary tables. The data were presented as mean ± SEM. Statistical evaluation was conducted by one-way ANOVA with repeated measures. *p* < 0.05 was considered significant.

## Results

### mRNA Expression of Group I Metabotropic Glutamate Receptors in Human and Rat Carotid Body

**FIGURE 1:**
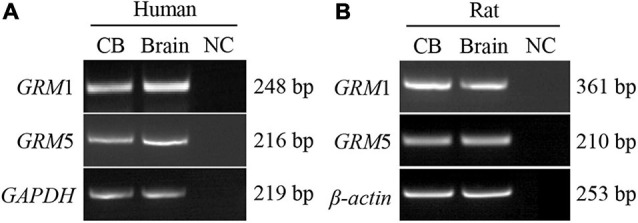
mRNA expression of group I mGluRs in human and rat CB. **(A,B)** Representative agarose gel images show the RT-PCR results of group I mGluR mRNA expression in human and rat CB. CB, left lanes in **(A,B)**. RNA extracted from the human brain cerebral cortex was used as positive control (brain, middle lanes in **A,B**). An equal volume of the negative cDNA control was used in the PCR as PCR negative control (NC; right lanes in **A,B**). GRM, glutamate metabotropic receptor.

Reverse transcription-PCR was performed to detect the mRNA expression of group I mGluRs (*GRM*1 and *GRM*5) in human and rat CB. As shown in Figure 1, PCR-amplified transcripts corresponding to the predicted sizes of *GRM*1 and *GRM*5 were detected in human (Figure 1A) and rat CB (Figure 1B) as well as brain cerebral cortex, which was used as positive control. These results indicate that both human and rat CB express all group I mGluR mRNAs.

### Protein Expression of Group I Metabotropic Glutamate Receptors in Rat Carotid Body

**FIGURE 2:**
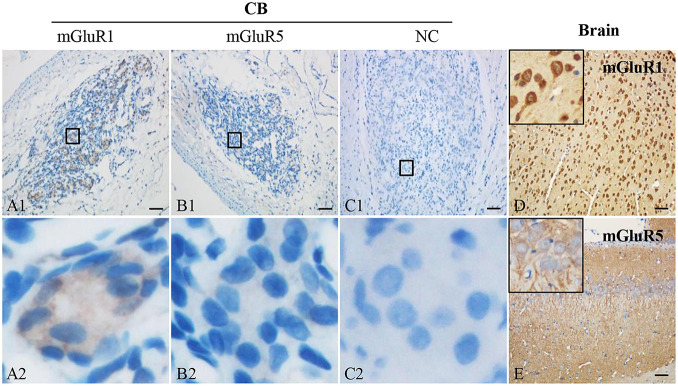
Immunohistochemical staining of mGluR1 and mGluR5 in rat CB. Representative images of rat CB and brain sections were immunostained with mGluR1 and mGluR5. Panels **(C1,C2)** are negative staining controls obtained by omitting the primary antibody. mGluR1 **(D)** or mGluR5 **(E)** staining of rat brain sections were used as positive controls. Panels **(A2–C2)** are higher-magnification images of framed areas in **(A1–C1)**, respectively. The brown staining represents the mGluR1 or mGluR5, while the blue staining represents hematoxylin-stained cell nuclei. mGluR, glutamate metabotropic receptor; NC, negative control. Scale bar, 50 μm for all images.

To detect the protein expression of group I mGluRs (mGluR1 and mGluR5) in rat CB, immunohistochemistry was performed. Representative photomicrographs in Figures 2A1,A2 showed that mGluR1-positive immunoreaction was ubiquitously detected in the clustering glomeruli of rat CB. However, mGluR5-specific immunostaining was not observed in rat CB (Figures 2B1,B2). The representative negative controls (NCs) in the absence of the anti-mGluR1 or mGluR5 antibody did not yield specific staining in rat CB (Figures 2C1,C2). Rat brain sections with intense immunoreactive mGluR1 (Figure 2D) or mGluR5 (Figure 2E) were used as positive controls. These results indicate that mGluR1 protein rather than mGluR5 protein is present in rat CB.

### Cellular Localization of Metabotropic Glutamate Receptors 1 in Rat Carotid Body

**FIGURE 3:**
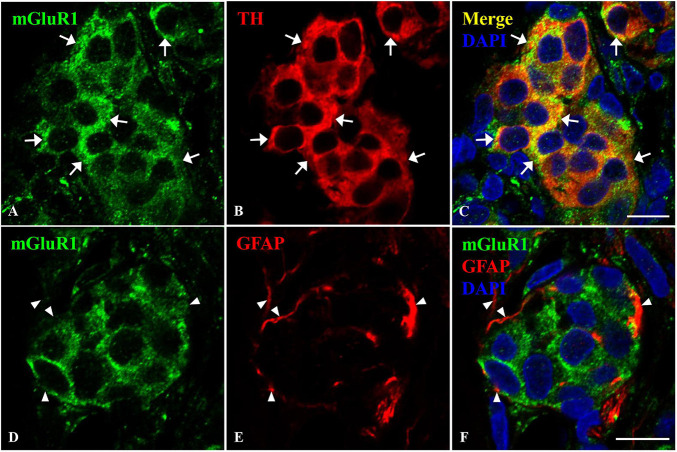
Distribution of mGluR1 in rat CB. Panels **(A–C)** are double immunofluorescence staining of mGluR1 (green) with TH (red) in rat CB. Panels **(D–F)** are double immunofluorescence staining of mGluR1 (green) and GFAP (red) in rat CB. Some TH-immunoreactive type I cells are immunoreactive for mGluR1 (arrows), whereas the GFAP-immunoreactive type II cells are not immunoreactive for mGluR1 (arrowheads). TH, tyrosine hydroxylase, the CB type I cells marker; GFAP, glial fibrillary acidic protein, the CB type II cells marker; mGluR, glutamate metabotropic receptor. Scale bar, 10 μm for all images.

The CB is composed of type I glomus cells and type II sustentacular cells. To further ascertain the localization of mGluR1 in rat CB, double immunofluorescence was performed. TH was used as the type I glomus cell marker and GFAP was used as the type II cell marker. As shown in Figure 3, the mGluR1 immunoreactive signal was diffusely present in the cytoplasm of round-shaped cells which are clustered in rat CB and co-localized with TH-positive immunoreactivity (Figures 3A–C), but not detected with GFAP-positive signals (Figures 3D–F), thus indicating that mGluR1 distributes in type I cells rather in type II cells in rat CB.

### Activation of Group I Metabotropic Glutamate Receptors Attenuates Carotid Body Response to Hypoxia

**FIGURE 4:**
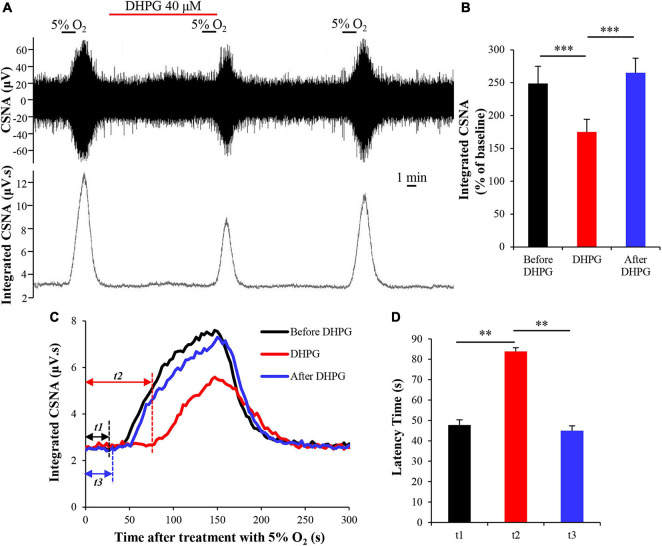
DHPG attenuates CB response to hypoxia. **(A)** Effect of DHPG (40 μM) on the CSNA under hypoxia conditions. Note that DHPG reversibly reduces the CSNA under hypoxia condition. **(B)** DHPG (40 μM) reduces the integrated CSNA during hypoxia. **(C)** Changes in integrated CSNA within 5 min of switching to 5% O_2_–5% CO_2_–90% N_2_ saturated Kreb’s solution. *t1*, latency time of CB response to hypoxia before treatment with DHPG (40 μM); *t2*, latency time of CB response to hypoxia while treatment with DHPG; *t3*, latency time of CB response to hypoxia after treatment with DHPG. **(D)** DHPG (40 μM) reversibly increases the latency time of CB response to hypoxia. DHPG, group I mGluRs agonist; CSNA, carotid sinus nerve activity; *t*, time. The data were presented as mean ± SEM. *n* = 9, ^∗∗^*p* < 0.01 and ^∗∗∗^*p* < 0.005 indicate significant difference (one-way ANOVA with repeated measures).

As a chemoreceptor, the CB senses the reduction of arterial PO_2_ during hypoxia, resulting in afferent activation of the CSN, which is also known as the CB response to hypoxia. To investigate the effect of mGluR1 on the CB response to hypoxia, CSN discharge was recorded from CB-sinus nerve preparations *ex vivo*. A representative trace of CSNA is shown in Figure 4A. Similar to a previous study (Rong et al., 2003), the baseline CSNA under 95% O_2_ was increased sharply after exposure to 5% O_2_ hypoxia. The time from exposure to hypoxia to the onset of CSNA increase is referred to as latency time (*t*; Figure 4C) of CB response to hypoxia. After application of selective group I mGluR agonist DHPG into basal perfusate (95% O_2_–5% CO_2_ saturated Kreb’s solution) for 10 min, DHPG (40 μM) significantly inhibited the CSNA under hypoxia conditions (normalized integrated CSNA before treatment with DHPG vs. normalized integrated CSNA while treatment with DHPG: 248.80 ± 26.17 vs. 175.03 ± 19.23%; *n* = 9, *p* < 0.005) (Figures 4A,B). The latency time of CB response to hypoxia was 47.78 ± 2.52 s before treatment with DHPG, 83.89 ± 1.82 s during treatment with DHPG, and 45.56 ± 2.36 s after treatment with DHPG (Figures 4C,D; *n* = 9, *p* < 0.01). These results indicate that group I mGluR activation inhibits CSNA under hypoxia and increases the latency time of CB response to hypoxia; that is, activation of group I mGluRs attenuates the CB response to hypoxia.

### Activation of Metabotropic Glutamate Receptors 1 Attenuates Carotid Body Response to Hypoxia

**FIGURE 5:**
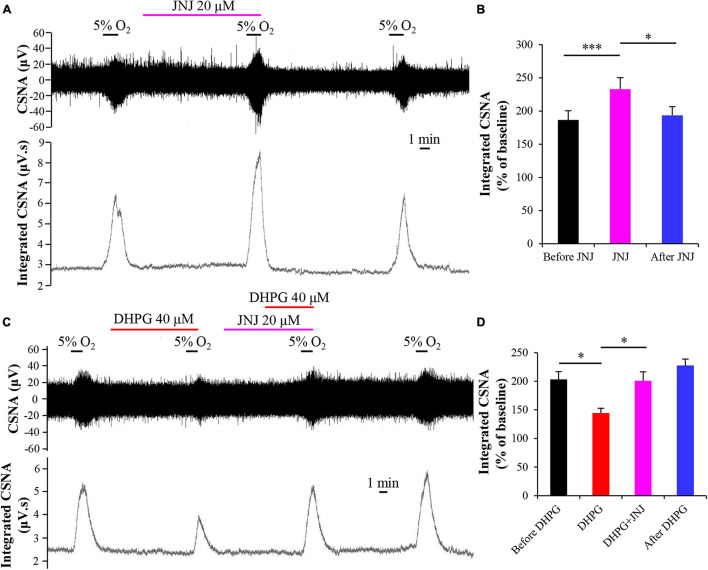
mGluR1 antagonist JNJ blocks the inhibitory effect of DHPG on CB response to hypoxia. **(A)** Effect of JNJ (20 μM) on the CSNA under hypoxia conditions. Note that DHPG reversibly enhances the CSNA under hypoxia conditions. **(B)** JNJ (20 μM) increases the normalized integrated CSNA during hypoxia. *n* = 7. **(C,D)** Pretreatment with JNJ prevents the inhibitory effect of DHPG on CB response to hypoxia. The data were presented as mean ± SEM. *n* = 4. ^∗^*p* < 0.05 and ^∗∗∗^*p* < 0.05 indicate significant difference (one-way ANOVA with repeated measures). CSNA, carotid sinus nerve activity; JNJ, JNJ16259685, mGluR1 antagonist.

To assess the role of mGluR1 in DHPG-attenuated CB response to hypoxia, we first investigated the effects of mGluR1 antagonist JNJ16259685 (JNJ) on CB response to hypoxia by applying JNJ (20 μM) in basal perfusate for 10 min before exposing it to hypoxia. We found that JNJ enhanced the CSNA under hypoxia conditions, as indicated by an increase in normalized integrated CSNA from 186.72 ± 13.72 to 232.77 ± 17.61% (*n* = 7, *p* < 0.005) (Figures 5A,B). The inhibitory effect of DHPG on CSNA under hypoxia conditions was also blocked by pre-perfusion with JNJ for 5 min, as the normalized integrated CSNA increased from 144.60 ± 8.05 (treatment with DHPG only) to 200.87 ± 15.77% (treatment with DHPG and JNJ) (*n* = 4, *p* < 0.05) (Figures 5C,D). These results indicate that DHPG attenuates CB response to hypoxia by activating mGluR1; that is, mGluR1 in CB may act as an inhibitory feedback regulator to modulate CB chemoreflex.

### Inhibitory Effect of (S)-3,5-Dihydroxyphenylglycine on Carotid Body Response to Hypoxia Involves the GABA_*B*_ Receptor

**FIGURE 6:**
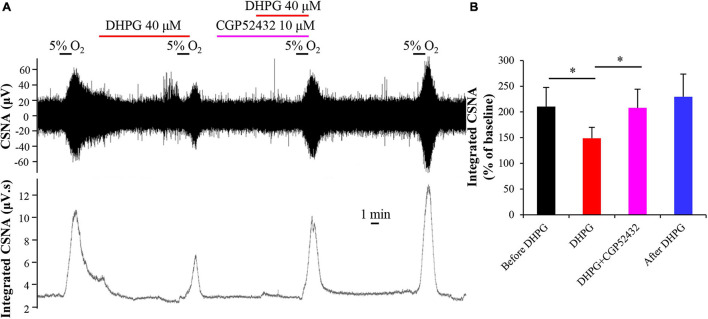
GABA_*B*_ receptor antagonist CGP52432 blocks inhibitory effect of DHPG on CB response to hypoxia. **(A)** Representative traces of changes in raw CSNA and integrated CSNA recordings. Note that pretreatment with CGP52432 prevents the inhibitory effect of DHPG on CSNA under hypoxia conditions. **(B)** Statistical analysis of normalized integrated CSNA after difference treatment. The data were presented as mean ± SEM. *n* = 5, ^∗^*p* < 0.05 indicates significant difference (one-way ANOVA with repeated measures). CSNA, carotid sinus nerve activity; CGP52432, GABA_*B*_ receptor antagonist; DHPG, group I mGluRs agonist.

Previous studies found that GABA inhibits hypoxia-induced postsynaptic sensory responses in coculture of rat CB type I cells with petrosal neurons by acting on GABA_*A*_ receptors (Zhang et al., 2009). We also found that GABA inhibits the rat CB hypoxia response *in vitro* (unpublished data). GABA also plays an autoreceptor feedback inhibition in CB response to hypoxia *via* acting on the GABA_*B*_ receptor in CB type I cells (Fearon et al., 2003), and the cross talk has been demonstrated between the GABA_*B*_ receptor and other receptors (such as GABA_*A*_ receptor, mGluR1, and NMDA receptor) (Xu et al., 2014). Therefore, we speculate that the GABA_*B*_ receptor may be involved in the DHPG-induced inhibitory effect on CB response to hypoxia by activating mGluR1. To determine whether activated mGluR1 exerts its inhibitory effect on CB response to hypoxia through GABA_*B*_ receptor, we applied the GABA_*B*_ receptor antagonist CGP52432 (10 μM) for 5 min before DHPG treatment. As shown in Figure 6, compared with normalized integrated CSNA during treatment with DHPG (148.96 ± 21.28%), the normalized integrated CSNA was increased to 208.00 ± 36.18% (*n* = 5, *p* < 0.05) after pre-perfusion with CGP52432. That is, CGP52432 could prevent the inhibitory effect of DHPG on CB response to hypoxia. This result indicates that the GABA_*B*_ receptor in CB may be involved in the mGluR1-induced feedback inhibition in CB response to hypoxia.

## Discussion

In this study, we found for the first time that mRNAs of group I mGluRs (mGluR1 and mGluR5) were expressed in human and rat CB, whereas the protein of mGluR1 instead of mGluR5 was detected in rat CB and scattered throughout the CB. Moreover, we determined that mGluR1 was distributed in type I glomus cells. Our investigation indicates that group I mGluR activation by DHPG inhibited CB response to hypoxia, while mGluR1 antagonist JNJ16259685 enhanced CB response to hypoxia and blocked the inhibitory effect of DHPG on CB response to hypoxia. We also found that the inhibitory effect of DHPG on CB response to hypoxia might be related to the GABA_*B*_ receptor in CB type I cells, as the inhibitory effect could be blocked by inhibition of the GABA_*B*_ receptor. Taken together, these results indicate that, rather than mGluR5, functional mGluR1 exists in the CB and may play a role in presynaptic feedback inhibition in rat CB hypoxia sensitivity.

The CB transmits the changes of oxygen levels in arterial blood through chemosensory synapses formed by type I cells (as presynaptic neuroendocrine cells) with CSN afferent fibers (as postsynaptic nerve endings). Hypoxia-induced depolarization of type I cells releases a variety of neurotransmitters, which are the material basis of CB oxygen sensory transmission (Ortega-Sáenz and López-Barneo, 2020), acting on the corresponding receptors of CSN afferent fibers. Studies have confirmed that neurotransmitters in CB type I cells such as ATP, ACh, 5-HT, and GABA regulate CB response to hypoxia by acting on P2X receptors (Zhang et al., 2000; Prasad et al., 2001; Iturriaga et al., 2007), nicotinic ACh receptors (nAChR; Zhang et al., 2000; Varas et al., 2003; Iturriaga et al., 2007), 5-HT_2_ receptors (Jacono et al., 2005), and GABA_*A*_ receptors (Zhang et al., 2009) on postsynaptic CSN afferent fibers, respectively. Nonetheless, the role of glutamate and its receptors, the most important neurotransmitter in the central nervous system, in CB response to hypoxia is unclear.

Torrealba et al. (1996) report that type I cells in the cat CB had high concentrations of glutamate, but glutamate release from *in vitro* cat CB superfusion with high potassium within 2 min was not observed. The perfusate used in this study was 50 or 70 mM potassium Krebs–Ringer–HEPES (KRH) solution. Accordingly, they speculated that glutamate could not be released as a neurotransmitter from type I cells. Interestingly, when Kim et al. (2004) measured the ACh release from rabbit CB, they found that 30 or 60 mM KCl stimulation for 15 min had no effect on ACh release, but increasing the KCl concentration to 100 mM significantly induced the ACh release from the CB, indicating whether or not a transmitter release from the CB triggered by high potassium *in vitro* might be dependent on potassium concentration and duration time. Hence, it cannot entirely exclude the possibility that the superfusion condition in the study by Torrealba was not optimum to induce glutamate release from the CB stimulated with high-potassium solution *in vitro*. Interestingly, Yokoyama et al. (2014) found that VGluT2 is localized in the carotid sinus afferent nerve terminals. Recently, we have demonstrated multiple subtypes of VGluTs (VGluT3) and EAATs (EAAT2 and 3) being present in the CB (Li et al., 2020). These data suggested that glutamate might be releasing from CB itself or carotid sinus afferent nerve terminals, and then acting as a neurotransmitter. We also found that functional NMDAR1 and AMPAR1 in rat CB might regulate the CB chemoreflex plasticity (Liu et al., 2009, 2018). In this study, we further discovered that mRNAs of group I mGluRs (mGluR1 and 5) were also expressed in human and rat CB (Figure 1); this finding is consistent with our previous report; i.e., the adrenal medulla, another oxygen sensor originating similarly with the CB, expresses both mGluR1 and 5 (Fan et al., 2020). Among group I mGluRs, only mGluR1 protein was detected in rat CB by immunostaining (Figure 2). Awobuluyi et al. (2003) reported that glutamate inotropic receptor NMDAR1 (NR1) mRNA was present in PC12 cells but not its protein, and the underlying mechanism might be from the 3′ untranslated region of NR1 blocking the translation of NR1 mRNA at initiation in PC12 cells. Therefore, rat CB expressing mGluR5 mRNA but lacking obviously expression of mGluR5 protein implies translational or posttranscriptional modification. Additional work will be necessary to characterize the discrepancy expression between mGluR5 mRNA and protein in rat CB.

As members of the G protein-coupled receptor family, mGluRs are divided into three subtypes, groups I, II, and III, based on its receptor structure and physiological activity (Ohashi et al., 2002). In the central nervous system, the three groups of mGluRs have different precise synaptic site locations. Generally, group I mGluRs are located primarily on the postsynaptic membrane, and groups II and III mGluRs are primarily on the presynaptic membrane (Shigemoto et al., 1997). However, some studies have shown presynaptic group I mGluRs. Romano et al. (1995) reported that some mGluR5 were located in the presynaptic axon terminal. Wittmann et al. (2001) also found that mGluR1 was localized on presynaptic elements of the excitatory synapses of substantia nigra pars reticulata by immunohistochemistry at the electron microscope level. In this study, mGluR1 immunoreactive signals were co-localized with TH immunoreactives, and moreover, both signals were diffusely present in the cytoplasm of clustered round-shaped cells (Figure 3), demonstrating the ubiquitous presence of mGluR1 in type I cells of rat CB. However, we cannot rule out that mGluR1 is also present in TH-positive nerve terminals projected from petrosal ganglion neurons, which has been shown to express TH (Czyzyk-Krzeska et al., 1991; Finley et al., 1992). Additional co-staining of mGluR1 with a specific marker of postsynaptic nerve ending, such as the P2X2 receptor, will help answer this question. Due to type I cells being presynaptic neuroendocrine cells to the afferent nerve terminal of CSN, we thus speculate that mGluR1 in the carotid chemoreflex might regulate the synaptic transmission *via* a presynaptic mechanism.

Group I mGluRs play an important role in synaptic transmission and synaptic plasticity through G protein coupling (Anwyl, 2009; Reiner and Levitz, 2018) or through non-canonical effector pathways (Eng et al., 2016). Previous studies demonstrated that group I mGluRs participated in pathogenic and adaptogenic response to hypoxia in rat brain development (Simonyi et al., 1999; Vetrovoy et al., 2021) and mGluR1/5 was involved in the enhancement of respiratory rhythm during early hypoxia by concomitant activation of L-type Ca(2+) channels in inspiratory brainstem neurons (Mironov and Richter, 2000). Our previous study also found that inhibition of mGluR1 blocked the hypoxia-induced activation of ERK1/2 in rat adrenal medulla (Fan et al., 2020). These data suggested that group I mGluRs are likely to be involved in hypoxic modification of channel activity or regulation of hypoxia response molecules in some conditions. In this study, we observed that superfusion of rat CB *in vitro* with DHPG, an agonist of group I mGluRs, decreased the acute hypoxia-evoked discharge activity of CSN (Figure 4), and this effect was blocked by mGluR1 antagonist JNJ16259685 (Figure 5), indicating an inhibitory effect of mGluR1 on CB response to acute hypoxia. There is evidence shown that, *via* activation of the G protein-coupled receptor downstream protein kinase C, postsynaptic mGluR1 mediates the potentiation of NMDAR activity in hippocampus glutamatergic synapses (Skeberdis et al., 2001) or the hyper-excitability of layer 5 pyramidal neurons in the rat anterior cingulate cortex (Gao et al., 2016). In contrast, some evidence showed that mGluR1 or 5 inhibits excitatory or inhibitory synaptic transmission in the hippocampus CA1 (Gereau and Conn, 1995; Manzoni and Bockaert, 1995) and the superior colliculus (White et al., 2003) *via* presynaptic mechanism. Studies also found that activation of group I mGluRs by DHPG induces a long-term depression of excitatory synaptic transmission in the hippocampus (Fitzjohn et al., 2001; Faas et al., 2002) or hippocampal interneurons *via* presynaptic P/Q-type Ca^2+^ channels (Le Duigou et al., 2011). Thus, activation of group I mGluRs results in different functions in the central and peripheral nervous systems, which depends on its localization on the synaptic site. Based on these above studies and our results in this study, we speculate that activation of mGluR1 likely reduces hypoxia-induced CB response by regulating the release of other neurotransmitters in presynaptic type I cells or by regulating P/Q-type Ca^2+^ channels in type I cells.

We also found that activation of mGluR1 by DHPG prolonged the latency time of CB response to hypoxia (Figures 4C,D). Hypoxia induces Ca^2+^-dependent release of a variety of excitatory (such as ATP and ACh) (Zhang et al., 2000) and inhibitory (such as GABA and dopamine) (Zhang et al., 2009, 2018) transmitters in rat CB. These transmitters, which released from rat CB, act on postsynaptic P2X2/3 receptors and nAChR to produce excitatory effects or on GABA_*A*_ receptors and D2 receptors to produce inhibitory effects on CSNA to regulate the final CSN afferent signal. Jacono et al. (2005) detected the release of neurotransmitters in the perfusion solution after treatment with acute hypoxia by using high-performance liquid chromatography coupled with an electrochemical detection system. They found that hypoxia induced the release of different neurotransmitters from type I cells in a time-dependent manner, i.e., different neurotransmitters released at different times following hypoxia. Therefore, we speculated that DHPG may inhibit the release of some neurotransmitters in CB type I cells under early hypoxia, subsequently leading to the prolonged latency of CB response to hypoxia.

Carotid body has a complex regulatory network. A variety of neurotransmitters or neuromodulators act on the corresponding receptors in type I cells or CSN afferent fibers to produce excitatory or inhibitory effects and ultimately regulate the input of CB type I cells’ oxygen-sensitive signals (Aldossary et al., 2020; Ortega-Sáenz and López-Barneo, 2020). Many of these receptors are G protein-coupled receptors located not only in CSN afferent fibers but also in type I cells and type II cells (Guidolin et al., 2018). For example, GABA inhibits hypoxia-induced release of ATP and ACh by acting on presynaptic GABA_*B*_ receptor-mediated activation of TWIK-related acid-sensitive potassium channel 1 (TASK-1) (Fearon et al., 2003). Evidence showed that G protein-coupled receptors may interact with each other under various physiological and pathological conditions (Guidolin et al., 2018, 2019). Based on a varietyof G protein-coupled receptors located in CB, the researchers suggested that there may be receptor–receptor interactions in CB (Porzionato et al., 2018). Previous studies found that there is also an interaction between GABA_*B*_ and mGluR1, which is not a direct physical interaction between both receptors but rather a synergistic enhancement effect between G protein-coupled receptors (Rives et al., 2009; Xu et al., 2014). In this study, our results showed that pre-blocking GABA_*B*_ receptor activity by superfusion with its antagonist CGP52432 at least partially blocked the inhibitory effect of DHPG on acute hypoxia-evoked discharge of CSN (Figure 6). This suggests that the inhibitory effect of mGluR1 activation on CB hypoxia response is at least partially related to the GABA_*B*_ receptor. Future studies will determine how mGluR1 activates GABA_*B*_ directly or indirectly.

**FIGURE 7:**
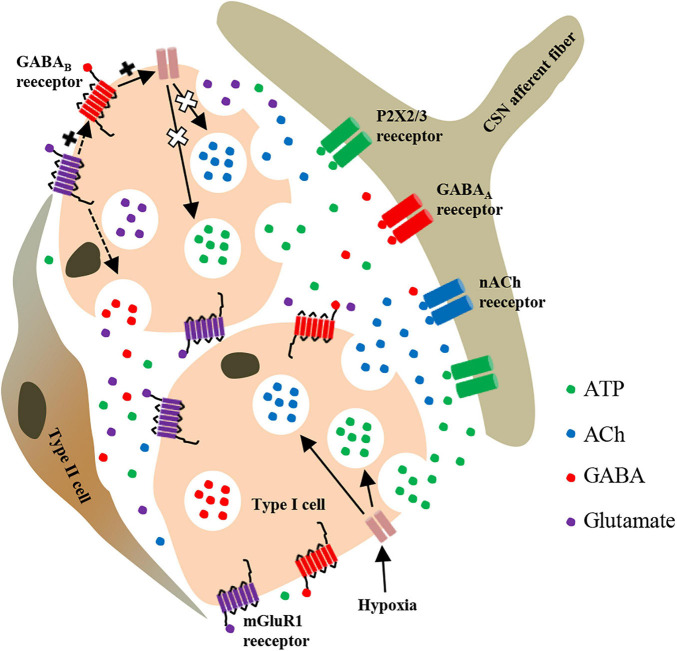
Schematic representation of mGluR1-mediated regulation of neurotransmitter release from rat CB type I cells during hypoxia. Inhibition of TASK-1 in type I cells by hypoxia leads to membrane depolarization and release of various neurotransmitters. Among these neurotransmitters, glutamate and GABA act on presynaptic mGluR1 and GABA_*B*_ receptors in themselves or in adjacent type I cells. GABA_*B*_ activation further activates TASK-1, thus leading to an increase of potassium outflow, then limiting the degree of depolarization of type I cells in order to inhibit the further release of ATP and ACh induced by hypoxia. Additionally, mGluR1 activation may enhance the activation effect of GABA_*B*_ receptor on TASK-1 through synergistic enhancement or modulate the release of GABA. In other words, both mGluR1 and GABA_*B*_ receptors on type I cells regulate the CSN afferent signals of CB response to hypoxia through a presynaptic feedback mechanism. P2X2/3, purinergic P2X2/3 receptor; nACh, nicotinic ACh receptor.

Taken together, our results in this study provide a novel candidate molecule that is involved in regulating CB response to hypoxia. We speculate that hypoxia not only stimulates the release of ATP, ACh, and GABA from CB type I cells but also stimulates the release of glutamate, which activates mGluR1 receptors on the surface of type I cells. Subsequently, mGluR1 may enhance the effect of GABA_*B*_ receptors by directly interacting with GABA_*B*_ receptors or indirectly modulating the release of GABA, and ultimately attenuates hypoxia-evoked enhancement of CSN afferent discharges (Figure 7).

Although this study found that GABA_*B*_ receptors may be involved in the inhibitory effect of mGluR1 on CB response to hypoxia, we cannot rule out other presynaptic mechanisms involved in this inhibitory effect. Further studies *via* single unit electrophysiological recording, along with primary coculture of type I cells with petrosal ganglion cells, will hopefully clarify the specific mechanism of mGluR1 on CB response to hypoxia. In addition, we cannot rule out that glutamate, like 5-HT (Jacono et al., 2005), participates in CB response to hypoxia as a modulator rather than an initiator. Therefore, further work on detecting glutamate release from CB after hypoxia treatment is necessary.

## Conclusion

In summary, our study found that the protein of mGluR1, rather than mGluR5, is mainly expressed in the CB and plays a presynaptic feedback inhibition on CB response to hypoxia.

## Data Availability Statement

The original contributions presented in the study are included in the article/Supplementary Material, further inquiries can be directed to the corresponding author.

## Ethics Statement

The studies involving human participants were reviewed and approved by the Ethics Committee of The First Affiliated Hospital of Xinxiang Medical University. The patients/participants provided their written informed consent to participate in this study. The animal study was reviewed and approved by the Institutional Animal Ethics Committee at The First Affiliated Hospital of Xinxiang Medical University.

## Author Contributions

YL designed the study. YL and BZ supervised the study execution. CL, CZ, and LH performed the experiments and analyzed the data. CL and YL wrote the manuscript. CL, BZ, and YL reviewed the manuscript. All authors read and approved the final manuscript.

## Conflict of Interest

The authors declare that the research was conducted in the absence of any commercial or financial relationships that could be construed as a potential conflict of interest.

## Publisher’s Note

All claims expressed in this article are solely those of the authors and do not necessarily represent those of their affiliated organizations, or those of the publisher, the editors and the reviewers. Any product that may be evaluated in this article, or claim that may be made by its manufacturer, is not guaranteed or endorsed by the publisher.

## Footnotes

1
https://www.ncbi.nlm.nih.gov/

## Abbreviations

ACh: acetylcholine
AMPARs: alpha-amino-3-hydroxy-5-methyl-4-isoxazole propionic acid receptors
CB: carotid body
CIH: cyclic intermittent hypoxia
CSN: carotid sinus nerve
CSNA: carotid sinus nerve activity
DHPG: (S)-3,5-dihydroxyphenylglycine
EAAT: excitatory amino acid transporter
GFAP: glial fibrillary acidic protein
iGluRs: ionotropic glutamate receptors
JNJ, JNJ16258685: (3,4-dihydro-2H-pyrano[2,3-b]quinolin-7-yl)-(cis-4-methoxycyclohexyl)-methanone
KARs: kainite receptors
GRM, mGluRs: metabotropic glutamate receptors
NC: negative control
NMDARs: *N*-methyl-D-aspartate receptors
nAChR: nicotinic ACh receptors
VGluT: vesicular glutamate transporter
PBS: phosphate-buffered saline
RT: reverse transcription
SD: Sprague-Dawley
TH: tyrosine hydroxylase
TASK-1: TWIK-related acid-sensitive potassium channel 1.
