# Supplementary material for: Metabotropic Glutamate Receptors 1 Regulates Rat Carotid Body Response to Acute Hypoxia via Presynaptic Mechanism
Source: Front Neurosci. 2021 Oct 5;15:741214. doi: 10.3389/fnins.2021.741214 (PMC8524001; doi:10.3389/fnins.2021.741214)
Supplement: Supplementary file 1 [file Data_Sheet_1.pdf]

Supplementary Material

Table S1 Normality tests of the data of Figure 4B in the manuscript

Tests of Normality			
	Shapiro-Wilk		
	Statistic	df	Sig.
Before DHPG	.841	9	.059
DHPG	.932	9	.499
After DHPG	.927	9	.450

Table S2 Normality tests of the data of Figure 4D in the manuscript

Tests of Normality			
	Shapiro-Wilk		
	Statistic	df	Sig.
t1	.886	9	.180
t2	.884	9	.172
t3	.912	9	.327

Table S3 Normality tests of the data of Figure 5B in the manuscript

Tests of Normality			
	Shapiro-Wilk		
	Statistic	df	Sig.
Before JNJ	.976	7	.939
JNJ	.907	7	.377
After JNJ	.976	7	.940

Table S4 Normality tests of the data of Figure 5D in the manuscript

Tests of Normality			
	Shapiro-Wilk		
	Statistic	df	Sig.
Before DHPG	.850	4	.225
DHPG	.907	4	.468
DHPG+JNJ	917	4	.522
After DHPG	.875	4	.319

Table S5 Normality tests of the data of Figure 6 in the manuscript

Tests of Normality			
	Shapiro-Wilk		
	Statistic	*df*	*Sig.*
Before DHPG	*.798*	*5*	*.078*
DHPG	*.854*	*5*	*.208*
DHPG+CGP52432	*833*	*5*	*.147*
After DHPG	*.833*	*5*	*.148*
